# The Impact of item flaws, testing at low cognitive level, and low distractor functioning on multiple-choice question quality

**DOI:** 10.1007/s40037-015-0212-x

**Published:** 2015-09-08

**Authors:** Syed Haris Ali, Kenneth G. Ruit

**Affiliations:** 1 Department of Physician Assistant Studies, University of North Dakota School of Medicine and Health Sciences, 501 N Columbia Rd., Stop 9037, 58202 Grand Forks, ND USA; 2 Educational Administration and Faculty Affairs, University of North Dakota School of Medicine and Health Sciences, Grand Forks, ND USA

**Keywords:** Assessment, Psychometrics, Validity, Medical education

## Abstract

**Background:**

This study investigated the impact of addressing item writing flaws, testing at low cognitive level and non-functioning distractors (< 5 % selection frequency) in multiple-choice assessment in preclinical medical education.

**Method:**

Multiple-choice questions with too high or too low difficulty (difficulty index < 0.4 or > 0.8) and insufficient discriminatory ability (point-biserial correlation < 0.2) on previous administration were identified. Items in Experimental Subgroup A underwent removal of item writing flaws along with enhancement of tested cognitive level (21 multiple-choice questions), while Experimental Subgroup B underwent replacement or removal of non-functioning distractors (11 multiple-choice questions). A control group of items (Group C) did not undergo any intervention (23 multiple-choice questions).

**Result:**

Post-intervention, the average number of functioning distractors (≥ 5 % selection frequency) per multiple-choice question increased from 0.67 to 0.81 in Subgroup A and from 0.91 to 1.09 in Subgroup B; a statistically significant increase in the number of multiple-choice questions with sufficient point-biserial correlation was also noted. No significant changes were noted in psychometric characteristics of the control group of items.

**Conclusion:**

Correction of item flaws, removal or replacement of non-functioning distractors, and enhancement of tested cognitive level positively impact the discriminatory ability of multiple-choice questions. This helps prevent construct-irrelevant variance from affecting the evidence of validity of scores obtained in multiple-choice questions.

**Electronic supplementary material:**

The online version of this article (doi:10.1007/s40037-015-0212-x) contains supplementary material, which is available to authorized users.

## Essentials

- The conceptual framework of validity requires analysis of difficulty and discriminatory ability of items given in an exam.
- Item flaws, testing of low cognitive function and low distractor functioning adversely affect the difficulty and discriminatory ability of multiple-choice questions used in assessment of basic medical sciences.
- Whether multiple-choice questions undergo removal of item flaws along with enhancement of tested cognitive level, or replacement or removal of non-functioning distractors, an improvement in their discriminatory ability with or without any significant change in their difficulty can be anticipated.
- This affirms the value of such interventions in enhancing the discriminatory ability, hence validity of scores, obtained on in-house multiple-choice assessment.

## Introduction

Assessment in undergraduate medical education is heavily reliant on multiple-choice questions. Quality of such in-house assessment has been reported as threatened because of lack of adequate training in the construction of multiple-choice questions [1]. Masters et al. [2] assessed multiple-choice assessments used in test banks accompanying selected nursing textbooks and found that around 47.3 % of the questions were written at low cognitive level of ‘plain factual recall’, and a meagre 6.5 % were written at the higher cognitive level of ‘analysis’. Jozefowicz et al. [3] reported on the quality of in-house exams in three US medical schools using a five-point scale and reported a mean rating of 4.24 for item writers trained by the National Board of Medical Examiners and 2.03 for those without any formal training. These works highlight the commonly perceived threats to the quality of multiple-choice assessments: item writing flaws and testing of lower cognitive function. Item writing flaws (Table 1) are violations of commonly accepted item writing guidelines meant to prevent test wiseness and irrelevant difficulty from influencing examinee performance on multiple-choice exams [4]. Downing reported that 10–15 % of students who failed in-house exams would have passed if flawed questions had been removed from the examinations [5]. In a study by Tarrant and Ware on assessment practices in nursing education [6], fewer examinees were found to pass the exams after post-hoc removal of flawed questions, and a greater number of examinees were found to score ≥ 80 % on unflawed questions than on flawed and unflawed questions combined. Such reports show that item flaws can surreptitiously increase both the pass as well as the failure rate in high-stakes exams.

**Table 1:** List of item writing flaws, published by the US National Board of Medical Examiners [4], with corresponding numerical codes used in this study

Code used for this study	Issues related to testwiseness
1	Grammatical cues—one or more distractors don’t follow grammatically from the stem
2	Logical cues—a subset of the options is collectively exhaustive
3	Absolute terms—terms such as ‘always’ or ‘never’ are in some options
4	Long correct answer—correct answer is longer, more specific, or more complete than other options
5	Word repeats—a word or phrase is included in the stem and in the correct answer
6	Convergence strategy—the correct answer includes the most elements in common with the other options
	*Issues related to irrelevant difficulty*
7	Options are long, complicated, or double
8	Numeric data are not stated consistently
9	Terms in the options are vague (e.g. ‘rarely,’ ‘usually’)
10	Language in the options is not parallel
11	Options are in a non-logical order
12	‘None of the above’ is used as an option
13	Stems are tricky or unnecessarily complicated
14	The answer to an item is ‘hinged’ to the answer of a related item

Although not classified as an item flaw, testing of lower (factual recall) rather than higher (application of knowledge) cognitive function has been considered a significant impediment to the quality of multiple-choice questions [7]. This is because clinical reasoning, and not plain regurgitation of facts, is required for sound application of basic medical sciences. Tarrant et al. [8] found that multiple-choice questions testing lower cognitive function were significantly more likely to contain item flaws than those testing higher cognitive levels. Newble [9] reported a greater difference in performance on free-response and multiple-choice versions of the same exams among medical students than among practising physicians and attributed this finding to testing of lower cognitive function, alongside greater reliance on guessing and cueing, offered by the multiple-choice questions. Several other scholars have discussed ways to effectively assess higher order thinking, clinical reasoning and problem-solving ability via careful construction of multiple-choice questions [10–13].

Another topic closely associated with quality of multiple-choice assessments is distractor functioning. A *functioning* distractor is an incorrect option that is selected by ≥ 5 % of examinees (i.e., ≥ 5 % selection frequency) and is selected more often by low-performing examinees than high-performing ones, which renders it a negative discriminatory ability [14]. On the other hand, a *non-functioning* distractor does not possess these desirable characteristics. Low distractor functioning has been reported to threaten the validity of scores obtained on multiple-choice questions [14, 15]. Using item-analysis data, Tarrant and Ware [16] eliminated the least selected distractor from 4-option multiple-choice questions and reported minimal impact on mean item difficulty and discriminatory ability. Similarly, in a seminal meta-analysis consolidating the findings from various studies, Rodriguez reported that eliminating the least functioning distractor caused no significant change in item difficulty and allowed the remaining distractors to exhibit greater selection frequency and discriminatory ability [14]. These reports show that elimination of rarely selected distractors may lend the benefit of reduced response time and increased content sampling for multiple-choice exams.

The study presented here asked the question, ‘What is the effect of correction of item writing flaws (including testing at a higher cognitive level) and removal or replacement of non-functioning distractors on difficulty and discriminatory ability of multiple-choice questions?’ Specifically, the authors were interested in the comparative benefit of these interventions. The conceptual framework used in this study was validity, as defined by Messick [17] and advanced by others [18–20], in which evidence from various sources is generated to support the meaning assigned to scores obtained on an assessment instrument. The source of particular interest in this study is difficulty and discriminatory ability of multiple-choice questions.

## Materials and methods

### Research design

A repeated-measures experimental research design was used. The study protocol was approved, and exempted from full review by the Institutional Review Board of University of North Dakota.

### Subjects

Two cohorts of Year 1 medical students (Cohort 1: *n* = 69, Cohort 2: *n* = 70) at the University of North Dakota School of Medicine and Health Sciences from the graduating class of 2016 and 2017 served as subjects. An expected, cohort-to-cohort difference in gender representation, undergraduate grade point average, and average medical college admissions test scores was seen amongst these cohorts.

The school’s medical education curriculum is a hybrid of patient-centred learning as well as traditional, discipline-based instruction. The multiple-choice exams used as the venue of this experiment are mandatory and are used to assess knowledge of the basic medical sciences at the end of each of the four 8-week curricular blocks during Year 1 (hence termed, ‘end-of-curricular block exams’). Assessment is criterion-referenced; students must score 75 % or more on an end-of-block multiple-choice examination to have their performance considered ‘satisfactory’. Since the revision of multiple-choice questions based on item analysis data, and venue of their administration (high-stakes, mandatory exams), is normal educational practice, and the study protocol was approved by the Institutional Review Board, subjects were not informed about the study.

### Procedure

Fifty-five multiple-choice questions with either too high difficulty (difficulty index < 0.4), too low difficulty (difficulty index > 0.8) or insufficient discriminatory ability (point-biserial correlation coefficient < 0.2) were identified from each end-of-block (Blocks I–IV) multiple-choice exam administered in the previous academic year. These questions represented a variety of preclinical subjects such as gross anatomy, physiology, biochemistry, pharmacology, genetics, developmental biology, and neuroscience.

### Intervention

The design of the study involved random placement of items (rather than subjects) in the experimental or the control group.

*Experimental Subgroup A* comprised 21 items that underwent correction of item writing flaws along with enhancement of tested cognitive level.

First, SHA studied the topics from recommended texts and removed the item flaws according to National Board of Medical Examiners guidelines [3]. Then, where needed, SHA developed a clinical vignette to assess knowledge of the same topic at a higher cognitive level. The clinical or laboratory vignette was added such that the item assessed application of knowledge and not just plain factual recall [21]. Cognitive level was enhanced only in those items in Experimental Subgroup A that previously tested plain factual recall. Table 1 displays a list of these flaws along with corresponding numerical codes used in this study.

KR provided input and further recommendations on item revisions proposed by SHA. Then, the item’s original writer was consulted and their consensus was sought on the changes. Upon approval from the item’s original author, the revised version of the item was administered in an end-of-curricular block exam during the next academic year. Table 2 displays a step-by-step elaboration on the intervention performed on items in Experimental Subgroup A.

**Table 2:** Example of the intervention (italics) performed on items in Experimental Subgroup A (removal of item writing flaws and enhancement of tested cognitive level) and Experimental Subgroup B (replacement of non-functioning distractors)

Before	After
Experimental Subgroup A Which of the following best describes the location of the prostate gland? A: Inferior and posterior to the neck of the bladder in the rectovesical pouch B: At the neck of the bladder superior to the pelvic diaphragm** C: At the neck of the bladder inferior to the pelvic diaphragm D: In the superficial perineal pouch E: In the deep perineal pouch *The topic of interest in this item was ‘location of the prostate gland’.* *The item was found to be testing low cognitive level owing to plain recall of a fact (i.e., location of the prostate gland).* *Moreover, the following flaws were identified in this item:* *a. Long or complicated options* *b. Non-logical order of options*	*SHA studied the topic ‘location of the prostate gland’ from recommended texts and developed the following clinical vignette to assess knowledge of the same topic at a higher cognitive level.* A 72-year-old male, in relatively good health, complains of frequent urination, weak stream, and post-void feeling of residual urine. Digital rectal exam reveals an enlarged organ. Which of the following describes the location of this organ? A: Deep perineal pouch B: Inferior to pelvic diaphragm C: Rectovesical pouch D: Superficial perineal pouch E: Superior to pelvic diaphragm** *Item flaw ‘long or complicated options’ was removed by simplifying the distractors. Note that the distractors underwent only simplification, and not removal or replacement with another distractor.* *Item flaw ‘non-logical order of options’ was removed by arranging the options in an alphabetical order.* *The item’s original writer was consulted who agreed with these changes. The revised version of the item was administered in the end-of-curricular block exam during the next academic year.*
Experimental Subgroup B A very premature infant is administered oxygen in the neonatal intensive care unit. Knowing that premature infants can also be cysteine-deficient, the patient is also given supplements of this amino acid to combat oxidative damage associated with oxygen toxicity. Cysteine is therapeutic because it is a precursor for what important intracellular antioxidant? A: Carnitine B: Glutathione** C: Histamine D: Phosphocreatine E: Serotonin *The topic of interest in this item is ‘Therapeutic basis of cysteine as an intracellular antioxidant’.* *Four distractors (A, C, D, E) in this item were found to be non-functioning (i.e., showed < 5 % selection frequencies)*	*SHA studied the topic ‘Therapeutic basis of cysteine as an intracellular antioxidant’ from recommended texts and developed four new distractors.* *A: Melatonin* B: Glutathione** *C: Uric acid* *D: Vitamin C* *E: Vitamin E* *The item’s original writer was consulted who agreed with the replacement distractors. The revised version of the item was administered in the end-of-curricular block exam during the next academic year.*

*Experimental Subgroup B* comprised 11 items. Nine of these eleven items underwent replacement, and two items underwent removal, of at least one non-functioning distractor. First, SHA identified non-functioning distractors based on item analysis data. Then, he studied the topics under assessment from recommended texts and developed new distractors based on his readings. KR provided input and further recommendations on possible distractor replacements. Then, the item’s original writer was consulted and their consensus was sought on the revisions. Upon approval from the item’s original author, the revised version of the item was administered in end-of-curricular block exam during the next academic year.

In the case of two items where an adequate replacement distractor could not be found (Supplementary material, Table 1, Items 31 and 32), the least functioning distractor was removed from the item. There were fewer items in this experimental group because some faculty members did not approve the replacement or removal of distractors from the items they had originally written. Also, the institutional policy required usage of multiple-choice questions with no fewer than four and no more than five options. Table 2 displays a step-by-step elaboration on the intervention performed on items in Experimental Subgroup B.

Revisions in all items in both Experimental Subgroup A and B were shared with the items’ original writers. Upon approval from the item’s original writer, revised items were administered, along with unrevised items, in end-of-curricular block exams over the next academic year. Despite initial plans, the authors were unable to keep the number of items evenly distributed among the experimental and control groups owing to variable extent of agreement on proposed revisions from items’ original writers.

Twenty-three items were randomly placed in the *Control group*. These items did not undergo any intervention, and were administered as-is in end-of-block exams over the next academic year.

### Data collection and analysis

The following data were collected for each item in the study.

- Item difficulty index before and after revision of the item. Difficulty index is the proportion of test-takers answering the item correctly (number of correct answers/number of all answers). Although there is no universally agreed-upon criterion, an item correctly answered by 40–80 % of the examinees (difficulty index 0.4–0.8) has been described as ‘moderately difficult’ [22]. For the purpose of this study, items with difficulty index of > 0.8 were classified as ‘too easy’, and those with difficulty index of < 0.4 were classified as ‘too difficult’.
- Item discriminatory ability, in the form of point-biserial correlation (also known as item-total correlation), before and after revision of the item. The point-biserial correlation coefficient measures the correlation between performance on an item (dichotomous variable [0 = incorrect, 1 = correct]) and overall performance on an exam (continuous variable) [23]. An item with point-biserial correlation < 0.2 is considered less helpful in separating high- and low-ability examinees and can be used to flag items for revision or removal [22, 23]. Point-biserial correlation was chosen for the purpose of this study, rather than biserial correlation or any other index, because of its ready availability from item analysis data, its prevalent use [14, 16], and reports that various indices of item discriminatory ability provide largely similar results [23, 24].
- The number of functioning distractors per multiple-choice question, before and after revision of each item. In this study, an incorrect option with ≥ 5 % selection frequency was considered a ‘functioning’ distractor in accordance with the criterion discussed in literature [14].
- Item-writing flaws in each item (Table 1).
- Cognitive level tested by the item. Level 1 was chosen for ‘plain factual recall’ and Level 2 was chosen for ‘application of knowledge’.

The collected data were stored in Microsoft Excel (2010) and analyzed via Microsoft Excel and SigmaStat v. 20.

## Results

### Experimental subgroup A

Along with removal of identified flaws, 14 of these 21 items also underwent enhancement of tested cognitive level (Supplementary material, Table 2, Column 3, cognitive level = 1).

Upon re-administration, an increase in the number of functioning distractors per multiple-choice question (from 0.67 to 0.81), a decrease in mean item difficulty index (from 0.86 to 0.85), and an increase in mean point-biserial correlation (from 0.05 to 0.19) was noted (Table 3). Among these 21 items, the number of items with moderate difficulty remained unchanged from 5. On the other hand, the number of items with adequate discriminatory ability increased from 0 to 10 (47 % increase); this increase was found to be statistically significant via Fisher’s exact test (two-tailed, *p* < 0.001) (odds ratio [OR] 39.26, 95 % confidence interval [CI] 2.10–732.36).

**Table 3:** Summary of psychometric characteristics before and after intervention in experiment and control group. Result of Fisher’s exact analysis is also shown in select cells

	Subgroup A (removal of IWFs + enhancement of CL)		Subgroup B (replacement or removal of NFDs)		Control group	
	Before	After	Before	After	Before	After
# of items	21	21	11	11	23	23
Average # of distractors per MCQ	3.62	3.62	3.73	3.55	3.48	3.48
Total # of distractors	76	76	41	39	80	80
Total # of FDs	14 (18 %)	17 (22 %)	10 (27 %)	12 (33 %)	23 (29 %)	22 (28 %)
Mean # of FDs	0.67	0.81	0.91	1.09	1	0.96
Average difficulty	0.86	0.85	0.85	0.8	0.84	0.83
Average pbi	0.05	0.19	0.04	0.19	0.05	0.06
# of MCQs with moderate difficulty	5	5	3	4 (9 % increase; df [1], *p* > 0.05)	10	12 (8 % increase; df [1], *p* > 0.05)
# of MCQs with sufficient discriminatory ability	0	10 (47 % increase; df [1], *p* < 0.05)	0	6 (56 % increase; df [1], *p* < 0.05)	0	0

*CL* cognitive level, *FDs* functioning distractors, *IWF* item-writing flaws, *MCQ* multiple-choice questions, *NFDs* non-functioning distractors, *pbi* point-biserial correlation.

### Experimental subgroup B

Items 31 and 32 underwent removal, rather than replacement, of the least functioning distractor.

Upon re-administration, an increase in the number of functioning distractors per multiple-choice question (from 0.90 to 1.09), a decrease in mean item difficulty index (from 0.85 to 0.80), and an increase in mean point-biserial correlation (from 0.04 to 0.19) was noted (Table 3). Among these 11 items, the number of items with moderate difficulty increased from 3 to 4 (9 % increase), which was found to be statistically insignificant via Fisher’s exact test (two-tailed, *p* < 0.001) (OR 1.52, 95 % CI 0.24–9.29). On the other hand, the number of items with adequate discriminatory ability increased from 0 to 6 (56 % increase); this increase was found to be statistically significant via Fisher’s exact test (two-tailed, *p* < 0.001) (OR 27.18, 95 % CI 1.28–574.35).

### Control group (C)

Upon re-administration, a decrease in the number of functioning distractors per multiple-choice question (from 1.00 to 0.96), a decrease in mean item difficulty index (from 0.84 to 0.83), and an increase in mean point-biserial correlation (from 0.05 to 0.06) was noted (Table 3). Among these 23 items, the number of items with moderate difficulty increased from 10 to 12 (8 % increase); this increase was found to be statistically insignificant via Fisher’s exact test (two-tailed, *p* = 0.768) (OR 1.41, 95 % CI 0.44–4.53). The number of items with adequate discriminatory ability remained unchanged from 0.

Similar flaws were discovered in the experimental and control group of items (Supplementary material, Tables 1, 2 and 3). The most common flaws were non-logical order of options (Flaw# 11), long, complicated or double options (Flaw # 7) and word repeats between the stem and correct answer (Flaw# 5). Other less common flaws seen in each group were tricky or unnecessarily complicated stems (Flaw# 13), collectively exhaustive subset of options (Flaw# 2), and long correct answer (Flaw# 4).

A peculiar finding was the disparity in pre-intervention tested cognitive level among the experimental and control group of items: 14 out of 21 Experimental Subgroup A items, 4 out of 11 Experimental Subgroup B items, and 21 out of 23 Control group (C) items were found at cognitive level ‘1’ (Supplementary material, Tables 1, 2 and 3, column 3). Since fewer Experimental Subgroup B items (36 %) were originally written at low cognitive level than the Experimental Subgroup A (67 %) or Control Group (C) (91 %) items, these groups were not equivalent in this regard. The possible effect of this finding on the outcome of interest (post-revision item difficulty and discriminatory ability) is discussed later.

## Discussion

The experimental study presented here addressed the impact of item flaws, testing of a lower cognitive function and low distractor functioning on the quality of multiple-choice questions used in in-house exams. The following are a few thoughts based on obtained results.

Firstly, an increase in the number of functioning distractors (≥ 5 % selection frequency) per item was noted after correction of item flaws (along with enhancement of tested cognitive level), as well as after replacement or removal of non-functioning distractors. An increase from 0.67 to 0.81 was noted in Experimental Subgroup A, from 0.91 to 1.09 in Experimental Subgroup B, while a slight decrease in the number of functioning distractors per item, from 1 to 0.96, was noted in the control (C) group of items (Table 3). Although this finding is promising, there is a lot of room for further improvement. One way to further increase the number of functioning distractors per multiple-choice question would be to give a free-response (fill-in-the-blank) version of the items to one cohort of students in a no-stakes setting, develop distractors from the most recurring incorrect responses which could be incorporated into the multiple-choice version, and administer the revised multiple-choice version of the items to subsequent cohorts. The authors of this study have used this method in a separate study with promising results.

The increase in number of functioning distractors per item noted from our interventions (0.81 from 0.67 in Experimental Subgroup A and 1.09 from 0.91 in Experimental Subgroup B) was similar to a study published by Tarrant and Ware [16] in which removal of the least functioning distractor increased in the number of functioning distractors per item from 1.32 to 1.49. We predict an even greater increase in the number of functioning distractors if an item undergoes *both* flaw removal (along with enhancement of tested cognitive level) and replacement or removal of non-functioning distractors. A future study can evaluate the cumulative effect of these interventions on psychometric characteristics of multiple-choice questions.

Secondly, removal of item flaws along with enhancement of tested cognitive level seems to have less of an impact on average item difficulty when compared with replacement or removal of non-functioning distractors. In our study, average item difficulty was increased by 1 % (difficulty index from 0.86 to 0.85) in Experimental Subgroup A and 5 % (difficulty index from 0.85 to 0.80) in Experimental Subgroup B (Table 3). This shows that replacement or removal of non-functioning distractors may increase item difficulty to a slightly greater extent than removal of item flaws (along with enhancement of tested cognitive level), and may be more helpful in constructing optimally difficult criterion-referenced examinations [20]. One interesting finding was the change in item difficulty noted in some Experimental Subgroup A items after removal of Item Flaw 11 (Options are in non-logical order) (Supplementary material, Table 2, Items 4, 7, 9 and 19). A quick explanation would be that removal of this flaw put the correct option at a position seldom selected by examinees, which impacted the difficulty of the revised version of the item. Or that examinee performance was influenced by a slight change in instructional emphasis (content of lectures or small group discussions) between different subject cohorts. However, upon a closer look, one can see that most of these items also underwent enhancement of tested cognitive level by inclusion of a clinical vignette. This might also explain the change in item difficulty noted after removal of such a seemingly small flaw.

Thirdly, both interventions improve the ability of multiple-choice questions to discriminate among high- and low-ability students. An average point-biserial correlation of 0.19 was observed post-intervention in both experimental subgroups (Table 3). This value (0.19) approximates the point-biserial correlation coefficient (≥ 0.2) recommended in psychometrics literature [20]. Moreover, both experimental subgroups experienced a significant increase in the number of items with recommended point-biserial correlation coefficients post-intervention (47 and 54 % increase, Experimental Subgroup A and B respectively) with no such change noted in the control group of items (Table 3). This implies that post-intervention performance on the experimental subgroup of items tended to be less influenced by factors other than examinees’ knowledge of content. For example, if some items in an exam have confusing stems or options (an item flaw), their difficulty may be irrelevant from examinees’ knowledge of content. This can weaken the evidence of validity of scores obtained on such exams. Downing and Haladyna describe this phenomenon as ‘Construct-Irrelevant Variance’, and have discussed its effect on meaningful interpretation of scores obtained on high-stakes exams [15].

There are a few limitations to the study’s findings. Firstly, the number of items used in this study was small, especially in those that underwent replacement or removal of non-functioning distractors (Experimental Subgroup B). Therefore, results obtained from study should be generalized with caution. Secondly, the pre-intervention level of tested cognitive level was found to be dissimilar among the experimental and control groups of items: 67 % Experimental Subgroup A items, 36 % Experimental Subgroup B items, and 91 % Control group (C) items were found to be testing low (Level 1) cognitive function (Supplementary material, Tables 1, 2 and 3; column 3). This disparity may account for some of the post-intervention findings in the experimental subgroups and may have confounded our results to some degree. In the future expansion of our study, we aim to evaluate the tested cognitive levels in advance and ensure that the experimental and control group of items are equivalent in this regard. Thirdly, neither removal of item flaws (along with enhancement of tested cognitive level) (Experimental Subgroup A), nor replacement or removal of non-functioning distractors (Experimental Subgroup B) resulted in a significant change in average item difficulty. One of the goals of our interventions was to bring the average item difficulty within a range of 0.4–0.8 to allow better discrimination amongst high- and low-ability examinees and greater reliability of obtained scores [20]. Perhaps simultaneous removal of item flaws (along with enhancement of tested cognitive level) and replacement or removal of non-functioning distractors may help increase item difficulty to a sufficient degree and further enhance the evidence of validity of obtained scores.

## Conclusion

The use of item analysis data in evaluating the quality of multiple-choice questions is highly beneficial in effectively assessing examinee knowledge on in-house exams. Addressing item flaws, tested cognitive level and distractor functioning requires a critical eye as well as considerable skill and resources. Moreover, faculty professional development in this area can be a challenging task. However, our results demonstrate that with removal or replacement of previously non-functioning distractors, or removal of item flaws along with enhancement of tested cognitive level, we can expect a significant improvement in our ability to discriminate between high- and low-ability examinees. This outcome is worth the effort, i.e. in-house exams that accurately identify truly competent learners who should progress to the next stage of training.

### Sources of support (grants) for this work

None.

## Electronic supplementary material

(DOCX 29 kb)
