# Supplementary material for: The Impact of item flaws, testing at low cognitive level, and low distractor functioning on multiple-choice question quality
Source: Perspect Med Educ. 2015 Sep 8;4(5):244–51. doi: 10.1007/s40037-015-0212-x (PMC4602009; doi:10.1007/s40037-015-0212-x)
Supplement: Supplementary file 1 — (DOCX 29 kb) [file 40037_2015_212_MOESM1_ESM.docx]

| **Supplementary tables**  Table 1. Experimental Subgroup B items. Flaw type, tested cognitive level (CL), # of functioning distractors (FDs), difficulty index (diff.) and point biserial correlation (pbi) before and after intervention. Items 31 and 32 underwent removal, rather than replacement, of the least functioning distractor. CL: 1 = plain factual recall, 2 = application of knowledge. |
| --- |

| Item ID | Flaw type | CL | Total distractors before | Total distractors after | FDs before | FDs after | Diff. before | Diff. After | pbi before | pbi after |
| --- | --- | --- | --- | --- | --- | --- | --- | --- | --- | --- |
| 22 | 5, 11 | 2 | 3 | 3 | 2 | 3 | 0.61 | 0.56 | 0.11 | 0.14 |
| 23 | 7; 11; 13 | 2 | 4 | 4 | 2 | 2 | 0.61 | 0.44 | 0.18 | 0.3 |
| 24 | 11 | 1 | 4 | 4 | 1 | 0 | 0.86 | 0.93 | -0.03 | 0.16 |
| 25 | 4; 7; 11 | 2 | 3 | 3 | 0 | 1 | 1 | 0.81 | 0 | 0.3 |
| 26 | 11 | 2 | 4 | 4 | 0 | 0 | 0.95 | 0.97 | 0.11 | -0.01 |
| 27 | 11 | 2 | 4 | 4 | 2 | 1 | 0.87 | 0.9 | 0.05 | 0.25 |
| 28 | 2, 11 | 1 | 4 | 4 | 0 | 1 | 0.97 | 0.91 | -0.1 | 0.01 |
| 29 | 11 | 1 | 3 | 3 | 0 | 0 | 1 | 1 | 0 | 0 |
| 30 | None | 2 | 4 | 4 | 0 | 1 | 0.96 | 0.7 | 0.06 | 0.28 |
| 31 | 11 | 2 | 4 | 3 | 2 | 2 | 0.68 | 0.79 | 0.04 | 0.41 |
| 32 | 11 | 1 | 4 | 3 | 2 | 1 | 0.87 | 0.81 | 0.02 | 0.25 |

| Table 2. Experimental Subgroup A items. Flaw type, pre-intervention tested cognitive level (CL), # of functioning distractors (FDs), difficulty index (diff.) and point biserial correlation (pbi) before and after intervention. CL 1 = plain factual recall, 2 = application of knowledge. | | | | | | | | | |
| --- | --- | --- | --- | --- | --- | --- | --- | --- | --- |
| Item ID | Flaw type | CL | Total distractors | FDs before | FDs after | Diff. before | Diff. after | pbi before | pbi after |
| 1 | 5; 11 | 2 | 4 | 0 | 0 | 0.95 | 0.93 | -0.03 | 0.19 |
| 2 | 11 | 2 | 4 | 0 | 0 | 1 | 1 | 0 | 0 |
| 3 | 11 | 2 | 4 | 0 | 0 | 0.96 | 0.97 | -0.08 | 0.06 |
| 4 | 11 | 1 | 4 | 2 | 0 | 0.86 | 0.99 | 0.08 | -0.04 |
| 5 | 11 | 2 | 4 | 1 | 1 | 0.91 | 0.85 | 0.16 | 0.49 |
| 6 | None | 1 | 4 | 1 | 0 | 0.87 | 0.96 | 0.08 | 0.17 |
| 7 | 11 | 1 | 4 | 0 | 1 | 0.93 | 0.77 | 0.02 | 0.08 |
| 8 | 11 | 2 | 4 | 1 | 1 | 0.89 | 0.93 | 0.12 | 0.29 |
| 9 | 11 | 1 | 4 | 1 | 1 | 0.91 | 0.84 | 0.06 | 0.38 |
| 10 | 5, 11 | 1 | 3 | 0 | 0 | 0.96 | 0.94 | 0.09 | 0.31 |
| 11 | 11 | 1 | 3 | 0 | 0 | 1 | 1 | 0 | 0 |
| 12 | 11 | 1 | 3 | 0 | 1 | 0.95 | 0.93 | 0.05 | 0.16 |
| 13 | 7; 13 | 1 | 4 | 2 | 3 | 0.55 | 0.43 | 0.08 | 0.17 |
| 14 | None | 1 | 3 | 0 | 0 | 0.93 | 0.97 | 0.09 | 0.2 |
| 15 | 13 | 1 | 3 | 1 | 1 | 0.66 | 0.45 | -0.06 | 0.01 |
| 16 | 7; 11 | 1 | 4 | 2 | 3 | 0.66 | 0.68 | -0.02 | 0.22 |
| 17 | 7; 11 | 1 | 3 | 0 | 0 | 1 | 1 | 0 | 0 |
| 18 | 11 | 1 | 3 | 2 | 3 | 0.55 | 0.5 | 0.12 | 0.23 |
| 19 | 11 | 1 | 4 | 0 | 1 | 1 | 0.87 | 0 | 0.39 |
| 20 | 11; 13 | 2 | 4 | 1 | 0 | 0.6 | 0.96 | 0.16 | 0.31 |
| 21 | 5; 7; 11; 13 | 2 | 3 | 0 | 1 | 0.97 | 0.87 | 0.13 | 0.39 |

| Table 3. Control group (C) items. Flaw type, tested cognitive level (CL), # of functioning distractors (FDs) and psychometric characteristics before and after intervention. CL: 1 = plain factual recall, 2 = application of knowledge. | | | | | | | | | |
| --- | --- | --- | --- | --- | --- | --- | --- | --- | --- |
| Item ID | Flaw type | CL | Total distractors | FDs before | FDs after | Diff. before | Diff. after | pbi before | pbi after |
| 33 | 2; 11 | 1 | 4 | 1 | 1 | 0.68 | 0.65 | -0.06 | 0.01 |
| 34 | 5; 11 | 1 | 3 | 2 | 3 | 0.61 | 0.56 | 0.11 | 0.14 |
| 35 | 11 | 1 | 4 | 1 | 0 | 0.86 | 0.93 | -0.03 | 0.16 |
| 36 | 11 | 1 | 4 | 0 | 0 | 0.98 | 0.97 | -0.02 | 0.09 |
| 37 | 4 | 1 | 3 | 3 | 2 | 0.58 | 0.68 | 0 | 0.18 |
| 38 | 11; 13 | 2 | 3 | 1 | 1 | 0.56 | 0.54 | 0.01 | 0.09 |
| 39 | 11; 13 | 1 | 3 | 2 | 2 | 0.58 | 0.58 | 0.12 | -0.07 |
| 40 | 11 | 1 | 3 | 0 | 1 | 0.98 | 0.89 | 0.01 | 0.07 |
| 41 | None | 1 | 4 | 1 | 1 | 0.89 | 0.9 | 0.13 | 0.05 |
| 42 | 11 | 1 | 3 | 1 | 1 | 0.85 | 0.9 | 0.11 | 0.14 |
| 43 | 11 | 1 | 3 | 1 | 0 | 0.88 | 0.9 | 0.15 | 0.13 |
| 44 | 11 | 1 | 3 | 0 | 0 | 0.97 | 0.97 | 0.1 | -0.02 |
| 45 | 11 | 1 | 4 | 1 | 1 | 0.92 | 0.93 | 0.01 | -0.07 |
| 46 | 11; 12 | 1 | 3 | 0 | 0 | 0.97 | 0.99 | 0.1 | -0.06 |
| 47 | 11 | 1 | 4 | 2 | 2 | 0.72 | 0.68 | 0.15 | 0.18 |
| 48 | 11 | 1 | 4 | 1 | 1 | 0.95 | 0.91 | 0.15 | 0.17 |
| 49 | None | 1 | 4 | 0 | 0 | 1 | 0.97 | 0 | -0.07 |
| 50 | 11 | 1 | 4 | 2 | 2 | 0.74 | 0.75 | 0.05 | 0.14 |
| 51 | 5 | 2 | 3 | 1 | 1 | 0.82 | 0.78 | 0.04 | 0.01 |
| 52 | 4; 11 | 1 | 4 | 0 | 0 | 1 | 1 | 0 | 0 |
| 53 | 11 | 1 | 4 | 2 | 2 | 0.82 | 0.79 | -0.07 | 0.03 |
| 54 | 11 | 1 | 3 | 0 | 0 | 1 | 1 | 0 | 0 |
| 55 | 11 | 1 | 3 | 1 | 1 | 0.92 | 0.86 | 0.15 | 0.14 |
